# 

**Published:** 1871-06

**Authors:** 


### INDEX OF SUBJECTS.

### ORIGINAL COMMUNICATIONS.

Gynæcological Medicine....................277
Hypodermic Medication....................286

### EXTRACTS AND GLEANINGS.

Epilepsy treated with Hydrate of Chloral—Aneurism cured by Hypodermic Injection of Ergotine—Nux vomica for Billousness, 295; Dropsy for Neuralgia Tetanus successfully treated by Hydrate of Chloral—Wakefulness—Epilepsy treated successfully by Assafœtida, 296; Diabetes treated by Carbonate of Ammonia—Carbolic Acid in Scarlet Fever—Corrosive Sublimate for improving the Constitution—Epithelioma successfully treated by Chlorate of Potash; Prophylactic action of Copper in Cholera Epidemics, 297; Internal administration of Chloroform—Condurango—Propylamine—a remedy for acute and chronic Rheumatism—Growth of the Nails as a Prognostic Indication in cerebral Paralysis. 293; Chronic Eczema—Acute Rheumatism—Relief of Pain in Otorrhœa, 299; Codeia and oxalate of cerium in Dysentery—Diptheria treated by Quinine—Croup treated by the vapor of slacking lime—Hydrate of Chloral in Singultus, 300; Tape-worm expelled by Bromide of Potassium—Idiopathic Anæmia—Granular Lids in Chronic Opthalmia—Arsenic in Dyspepsia—Chloral in rigid os uteri, 301; Hypodermic doses of Morphia—Treatment of Lumbago—Local application to Burns—Glycerate of Tar, 302; Suppurative Hepatitis successfully treated with Muriate of Ammonia—Chlorosis, 303; New Operation for Entropium—Turpentine in Infantile Convulsions—Neuralgia—Intussusceptions treated by Injections of warm water—Syphilitic Ulcers, 304; Antagonistic Powers of Opium and Belladonna—Hydrochlorate of Apomorphia in rigid os—Diarrhœa of children—Ether in Flatulence—Toothache, 305; Purgatives in Skin Diseases—Tea-leaf poultices for Burns—Curative effects of Pregnancy on Prolapsus Uteri—New Emmenagogue—Post Partem Hemorrhage, 306; Secondary Syphilis—Intermittent Fever—Diabetes—Convulsions caused by blows upon the head, 307; Corrective influence of Bromide of Potassium over Opium—Cancrum oris—Sub nitrate bismuth in Cholera Infantum—Bromide of Iron in Involuntary Seminal Emissions and Spermatorrhœa, 308; Burns and Scalds—Chorea treated by ether spray to the spine—Iron in miasmatic diseases—Granular Lids—Poultices—Neuralgia accompanying Shingles, 309; Strychnia in the advance stages of Typhoid Fever—Carbolic Acid in Flavus and Crusta Lactea—Chronic Rheumatism—Staphisagria—Bromide of Potassium in Nettle Rash, 310; Camphor, etc., 311; Varicose Ulcers—Chloral in Cerebro-Spinal Meningitis—Bromide of Iron in Tertiary Syphilis—Nitro-Muriatic Acid in Urticaria—Colocynth, 312; Chloral in Obstetrics—Spongio Piline—Cure of Fistula in Ano without the Knife—Hypodermic injection of morphia in cholera morbus and dysentery, 313; Dysentery treated by Ipecac—Iodoform Ointment—Ascites successfully treated by oil of Copabia—Nux Vomica, 314; Lobelia—Cannabis Indica—Diagnosis of Pain, whether Rheumatic or Neuralgic, 315.

### CORRESPONDENCE—HYGENIC AND MISCELLANEOUS.

Our Corolina Correspondent....................316
Letter from a Correspondent....................317
The Mortality of Infancy....................320
Analysis of Hair Tonics and Restoratives....................327

### EDITORIALS, REVIEWS, ETC.

Georgia Medical Society of Savannah....................328
Our Exchanges....................332
The American Medical Association—Call for a Convention of Physicians....................334
Dr. S. C. Hitchcock—Case of Dr. W. G. Drake....................335

